# P02.115. Optimism and stress: how cultivating a positive view on the future can lead towards reduced basal stress and a more adaptive stress response

**DOI:** 10.1186/1472-6882-12-S1-P171

**Published:** 2012-06-12

**Authors:** Y Meevissen, M Peters

**Affiliations:** 1 Maastricht University, Maastricht, Netherlands

## Purpose

Optimism, a personality trait defined as holding general positive beliefs regarding the future, has been associated to improved physical health and longevity. One of the underlying mechanisms may be a reduced physiological stress response. Previous studies found that optimism was related to a lower blood pressure response to experimental stress and to a lower cortisol awakening response (CAR). It remains to be determined whether optimism per se, the associated difference in health behaviors (e.g. physical activity, eating and sleeping behavior) or another third variable is responsible for attenuating the physiological stress response. The aim of the present study was to clarify the causal status of optimism in relation to cortisol responsivity to stress.

## Methods

Optimism was experimentally induced by having participants construct and visualize their best possible self (BPS) over a course of 2 weeks. We previously showed that a BPS intervention leads to sustained increases in optimism. A matched control group engaged in a 2-week time management training. Before and after the BPS or control intervention, cortisol assessment in daily life was performed on two consecutive days and the CAR was examined. In addition, all participants were administered the TSST before and after the intervention and stress induced cortisol was examined.

## Results

Both groups showed a decrease in CAR after the intervention. Only the BPS group showed a pre to post intervention decrease in the cortisol response and a faster recovery towards baseline after the TSST.

## Conclusion

The present study gives evidence for the causal influence of optimism on the physiological stress response. Cultivating a positive view on the future reduces basal stress levels and creates a more adaptive stress response.

